# Posttraumatic growth theory as a framework for heart failure self-care intervention design

**DOI:** 10.3389/fpsyg.2026.1699377

**Published:** 2026-04-15

**Authors:** Rebecca Meraz, Gary Elkins, Jocelyn Shealy McGee, Bret A. Moore

**Affiliations:** 1 Louise Herrington School of Nursing, Baylor University, Dallas, TX, United States; 2 Department of Psychology and Neuroscience, Baylor University, Waco, TX, United States; 3 Garland School of Social Work, Baylor University, Waco, TX, United States; 4 Boulder Crest Institute, Bluemont, VA, United States

**Keywords:** adherence-compliance-persistence, conceptual model, heart failure, intervention design, posttraumatic growth (PTG), psychological growth, self-care

## Abstract

**Background:**

Heart failure (HF) is a life-altering diagnosis that requires sustained self-care and adherence to complex treatment regimens, yet patients may struggle with the psychological burden of managing a progressive, life-altering illness. Although positive psychological approaches are gaining attention in cardiovascular care, growth-oriented frameworks remain underexplored. Posttraumatic Growth (PTG) theory, which describes positive psychological change emerging through engagement with highly challenging life circumstances, may offer a novel conceptual lens for understanding HF self-care and guiding intervention development.

**Methods:**

This paper introduces PTG theory as a novel theoretical lens for understanding psychological adaptation and adherence in HF self-care. By extending PTG, a framework previously unused in HF, to the chronic demands of daily self-care, we propose new explanatory pathways linking growth-oriented processes to potential sustained adherence. To illustrate the framework’s applicability, we draw on two selected example cases from a small, early-stage positive psychology program development effort. These illustrative cases informed the development of a proposed PTG-informed conceptual model, CardioWell.

**Results:**

The illustrative cases contained examples of processes aligned with all five PTG domains: personal strength, spiritual and existential change, improved relationships, appreciation of life, and new possibilities. These examples suggest how engagement in growth-oriented psychological processes might support coping with the ongoing demands of HF self-care. By identifying psychologically meaningful targets and proposing conceptual pathways linking PTG-related processes to HF self-care, this work provides a strong theoretical foundation for the PTG-informed CardioWell model.

**Conclusion:**

This paper advances PTG theory as a novel theoretical lens for understanding psychological adaptation in HF self-care and for guiding the development of growth-oriented interventions. A PTG-informed conceptual model (CardioWell) is presented to illustrate how growth-oriented psychological processes could be targeted in future intervention development. These proposed pathways are exploratory and warrant formal evaluation in future research.

## 1 Introduction

Heart failure requires continual, long-term symptom management through self-care, which includes monitoring bodily symptoms, weighing daily, exercising, eating a low salt diet, and adhering to prescribed medications (Moser et al., 2012). Despite the proven health benefits of self-care practices, such as reduced hospitalizations and mortality (Calero-Molina et al., 2022), more than half of patients struggle to adhere to treatment regimens (Heo et al., 2021). Many patients struggle with adherence due to psychological distress, the burden and rigor of their personalized regimen, and diminished resilience (Liljeroos et al., 2020; Meraz et al., 2023d).

Existing HF self-care interventions often emphasize education and deficit-based behavioral strategies, such as skills training, reminder systems, or counseling techniques (Toukhsati et al., 2019; Lancey and Slater, 2024; Longhini et al., 2025). Although interest in positive psychological health in shaping health behaviors is increasing (Celano et al., 2018; Feig et al., 2022), less attention has been given to psychological growth-oriented frameworks that may help individuals engage meaningfully with the difficulty of HF self-care. Posttraumatic growth (PTG) theory (Tedeschi and Calhoun, 1996, 2004), which describes positive psychological change arising through sustained struggle with highly challenging life circumstances, may offer a novel theoretical lens for understanding HF self-care adherence and for guiding the development of growth-oriented interventions.

To our knowledge, PTG theory has not been applied to HF self-care. The purpose of this paper is therefore to introduce PTG theory as a novel theoretical lens for HF self-care adherence and to propose potential explanatory pathways within a conceptual intervention model, CardioWell. To illustrate this theoretical extension, we draw on two selected narratives from a small, non-PTG-based positive psychology program development effort. We map the five PTG domains onto the psychological challenges of HF self-care to demonstrate how the daily struggle of medication adherence, symptom monitoring, diet, and exercise may serve as catalysts for psychological growth. This extension of PTG theory opens new explanatory pathways and informs the development of the CardioWell conceptual model.

## 2 Posttraumatic growth theory

Posttraumatic growth, first identified and defined by Tedeschi and Calhoun (1996, 2004), proposes that positive psychological change can develop through the process of struggling with traumatic experiences that challenge an individual’s core assumptions about the world, self, and expectations for the future. When familiar ways of coping no longer suffice, individuals are pushed into a period of intense reflection, reevaluation, and meaning reconstruction. According to PTG theory, growth emerges as an individual actively engages with the trauma to discover new strengths, appreciation for life, existential and spiritual understandings, relational connections, and new possibilities for living (Tedeschi and Calhoun, 2004). Rather than returning to baseline, individuals who experience PTG adapt to their circumstances and develop enhanced perspectives and capabilities that represent meaningful progress beyond where they were before the challenging experience. For most, the experience of PTG is deeply profound and has a lasting impact on functioning and life goals (Tedeschi and Calhoun, 2004).

There are five domains of PTG that represent positive changes that individuals may experience as a result of their struggle with adversity:

- *Personal Strength*: recognition and development of personal strengths for emotional regulation, developing a stronger sense of resilience, identity, and capacity to manage challenges.
- *Spiritual and Existential Change*: growth may manifest as increased connection to something greater than oneself and deeper engagement with questions about purpose, mortality, and existence. Within PTG theory, it is proposed that individuals may transform their struggle and develop a new or deeper sense of meaning and understanding related to spiritual, religious, and existential themes.
- *Improved Relationships*: greater awareness of important relationships in one’s life, development of more empathy and compassion toward others, and increased valuing of relationships lead to stronger social connections and more meaningful relationships with others that result from the struggle with adversity.
- *Appreciation of Life*: greater appreciation of life can involve a changed sense of priorities and greater gratitude for life, deeper awareness about daily experiences, and a fundamental shift in how they perceive the value of being alive.
- *New Possibilities*: as an individual experiences PTG, it can lead to awareness of new directions for their life, openness to new life paths or goals, and consideration of new interests.

PTG is theorized to emerge from engagement with experiences that are highly distressing, threatening, and disruptive to an individual’s assumptive world (Tedeschi and Calhoun, 2004). The life-altering trauma forces individuals to question their assumptions about themselves, the world, and what the future may bring. The first step toward PTG is cognitive engagement with adversity to make sense of the experience and its meaning (Why did this happen? What does this mean? What now?). Next is cognitive processing, where individuals reconsider basic assumptions about themselves (What am I made of? What are my strengths?), their relationships, and the world around them. The potential for PTG is increased when cognitive processing involves deliberate rumination (i.e., intentional, constructive reflection on the experience and its meaning) rather than intrusive rumination (i.e., involuntary, repetitive, and distressing thoughts about the trauma). Growth is further supported through emotional disclosure, adaptive coping strategies (e.g., acceptance, re-anchoring of life purpose, problem-solving), the mobilization of resilience resources, and positive reframing (Henson et al., 2021).

Although PTG theory was initially developed to understand adaptation following discrete, “seismic events,” subsequent scholarship has emphasized that traumatic experiences may also be chronic, cumulative, or progressively destabilizing over time (Hamama-Raz et al., 2021; Gendre et al., 2025). Chronic, life-threatening illnesses can function as seismic disruptions when they shatter core assumptions about self-worth, personal capabilities, bodily integrity, and the benevolence and predictability of the world and people (Sawyer et al., 2010; Hamama-Raz et al., 2021; Pięta and Rzeszutek, 2022). HF exemplifies this form of chronic trauma through unpredictable and life-threatening exacerbations, progressively debilitating symptoms, and irreversible illness, generating cumulative adversity and psychological distress (Di Giacomo et al., 2025). The daily demands of HF self-care intensify distress by requiring patients to repeatedly confront their vulnerability, mortality, and increasing dependency on others. Together, the chronic nature of HF and the unrelenting burden of performing self-care tasks produce a life-altering disturbance that PTG theory identifies as fertile ground for psychological growth.

## 3 Conceptualizing PTG theory in heart failure self-care adherence

Daily HF self-care is clinically necessary but also a continual challenge (Hobbs, 2002; Thyagaturu et al., 2025). HF self-care encompasses a set of daily behaviors aimed at preventing fluid overload, minimizing acute exacerbations, and maintaining clinical stability. Self- care tasks include strict medication adherence (especially diuretics), daily weights, symptom monitoring, maintenance of a low-sodium diet, engagement in physical activity, and prompt reporting of worsening symptoms to clinicians (Moser et al., 2012; Jaarsma et al., 2021). Studies consistently demonstrate that patients perceive HF treatment and the daily self-care workload as highly physically, psychologically, and socially burdensome (Meraz, 2020; Nordfonn et al., 2020; Meraz et al., 2023d, 2025; Smith et al., 2025). Accordingly, the higher the perceived stress of treating and managing HF symptoms, the lower the self-care adherence (Smith et al., 2025).

Research to date on PTG interventions has mainly focused on individuals who have been exposed to some profound psychological trauma, such as seen among military veterans and first responders (Mark et al., 2018; Donovan, 2022). This research has shown that an intervention based on PTG theory can have a large impact on more effective coping with adversity, greater appreciation of life, gratitude, new personal strengths, deeper personal relationships, and finding meaning and purpose (Moore et al., 2021; Rhodes et al., 2024). A growing body of evidence among recovered cancer patients also suggests that PTG interventions can be highly effective in increasing resilience, health, and well-being (Vrontaras et al., 2023). However, PTG theory has yet to be applied to patients struggling with managing HF.

It is through daily HF self-care that many of the challenges to core assumptions of self, others, and the world surface. For example, patients may struggle to reconcile their former self with their current limited capacity, especially when they question their ability, inner strength, or willpower to perform self-care tasks (Riegel et al., 2022). Relationships with support systems may be strained as patients worry that their self-care obligations are burdensome to caregivers or question their trust in others to provide necessary support (Chung et al., 2013; Meraz et al., 2023d). For some, performing difficult and stressful self-care routines that do not lead to a cure leads to existential questioning about the meaning and purpose of continuing difficult self-care routines (Sethares et al., 2021). The struggle to adhere to self-care is often compounded by declining physical health and low mood, including depression and anxiety (Meraz et al., 2023d, 2025; Platz et al., 2025). Furthermore, particular HF medications such as diuretics are often perceived as highly stressful, as the resulting frequent and excessive urination impairs travel, social engagement, uninterrupted sleep, and overall quality of life (Meraz et al., 2023d, 2025).

Together, these insights suggest that HF self-care can be a persistent, identity-disrupting experience that continually challenges patients’ assumptions about their bodies, capabilities, and future. Because PTG theory explains how individuals can derive meaning, strength, and renewed purpose from this kind of ongoing disruption, it offers a compelling framework for understanding how HF patients may transform the burdens of self-care into opportunities for psychological growth. The following discussion examines how well-documented struggles in HF self-care are aligned with weaknesses across the five domains of PTG, highlighting why a growth-oriented framework may be particularly well suited to this population.

### 3.1 Personal strength and heart failure self-care

Evidence consistently demonstrates that HF self-care adherence is substantially impacted by internal motivations (Masterson Creber et al., 2017; Meraz et al., 2023a), positive or negative self-perceptions (Alvarez et al., 2016; Riegel et al., 2017), and levels of self-efficacy (Huang et al., 2022; Riegel et al., 2022). Collectively, this research highlights that HF patients frequently confront identity-related dilemmas in the face of ongoing self-care demands, which directly maps to the PTG domain of personal strength. Patients describe questioning whether they have the inner strength to sustain unremitting self-care demands or the capacity to perform self-care routines in the face of significant physical and emotional strain (Meraz et al., 2023d). For some patients, the struggle of self-care adherence erodes their sense of self, prompting harsh self-judgements that recast nonadherence as laziness, moral failure, or stubbornness (Meraz et al., 2025).

Although resilience is recognized as the capacity to persevere by positively adapting to difficult life circumstances, many patients with HF struggle to access or sustain this resource. Low levels of resilience have been linked to worse medication and HF self-care adherence, further compounding the challenges of HF management (Meraz et al., 2023c; Van Rijn et al., 2023). When resilience is low, hopeful states are diminished, and patients remain vulnerable to discouragement and disengagement from their self-care regimen (Meraz et al., 2023a,b,c,d). A growing body of evidence suggests that a positive outlook (i.e., gratitude, hopefulness, meaning in life), adaptive coping, and social support build resilience for sustaining HF self-care adherence during stress (Meraz et al., 2023a,b,c,d, 2025). But without supportive mechanisms to bolster resilience, the ongoing burden of HF self-care may continue to erode patients’ motivation and capacity to adhere over time. PTG-informed strategies that reframe the hardship of self-care as an opportunity for growth may enable patients to recognize inner strengths unearthed through adversity and to view themselves as fully capable of attaining and sustaining their HF self-care goals.

### 3.2 Meaning-making and existential distress in heart failure self-care

Accumulating evidence has shown that the overwhelming burden of HF self-care can pose existential challenges that impact a sense of meaning in life and purpose (Caboral et al., 2012; Kim et al., 2019), resulting in hopelessness and higher rates of self-care nonadherence (Meraz et al., 2023c; Mehri et al., 2024). Patients question the point of continuing difficult, lifestyle-altering self-care regimens and enduring treatments that do not make them feel better or improve their quality of life (Meraz et al., 2025). This lack of perceived purpose substantially undermines motivations to adhere (Riegel et al., 2022). The existential questioning reported by patients with HF mirrors meaning crises seen with trauma. Prior work has demonstrated that a higher sense of purpose and meaning in life can foster better HF self-care and buffer against the cardiotoxic effects of stress (e.g., inflammation, increased heart rate, and blood pressure)(Kim et al., 2019). Because PTG theory specifically targets meaning-making as a way to transform adversity into motivation to grow and achieve new goals, a PTG-based intervention could help patients reframe self-care as a meaningful act, thereby helping them to perceive self-care as less difficult and fostering a sense of purpose for adherence.

### 3.3 Deeper personal relationships vs. lack of social support in heart failure patients

Researchers agree that social isolation and a decline in personal relationships are drivers of problems with HF self-care adherence (Fivecoat et al., 2018; Irani et al., 2019; Meraz et al., 2023b; Babygeetha and Devineni, 2024). In the context of HF, social support encompasses emotional, instrumental (practical assistance with self-care), and informational support received/perceived from family members, social networks, caregivers, and other meaningful relationships, such as spiritual companions (Fivecoat et al., 2018; Carney et al., 2020; Babygeetha and Devineni, 2024). Left to manage HF without adequate support has been reported by patients as intensely stressful and burdensome (Meraz et al., 2025). Conversely, patients with adequate social support report high levels of self-care confidence and better overall self-care (Fivecoat et al., 2018). Within the PTG framework, addressing inadequate social support for self-care adherence through relational growth can strengthen relationships that buffer stress and support adaptive coping, thereby creating new avenues for assistance and support.

### 3.4 Appreciation in life and psychological affect in heart failure

Psychological distress is pervasive in the self-care experiences of patients with HF. Depression and anxiety are significantly more prevalent in patients with HF compared to the general population. Recent estimates demonstrate that almost half of all patients with HF have significant clinical depression or anxiety (Rashid et al., 2023). It is well established in the cardiovascular literature that individuals experiencing psychological distress, such as depression and anxiety, are less motivated to adhere to their self-care recommendations, including medication adherence (Moser et al., 2016; Ferguson et al., 2017; Celano et al., 2020). Studies reveal that patients may fear losing quality of life, bodily control (e.g., medication side effects), independence, autonomy; dread urinary accidents from diuretic therapy that threaten their dignity; worry about overburdening their caregivers; and experience persistent anxiety and uncertainty about recognizing and managing life-threatening symptoms (Meraz, 2020; Sethares et al., 2021; Meraz et al., 2023d, 2025). Because psychological distress is so pervasive in HF, the PTG domain of appreciating life may be particularly critical for supporting their capacity to cope with ongoing self-care demands.

Emerging evidence about the role of positive psychological states in HF self-care suggests that positive emotions, including gratitude, hope, and optimism, significantly support improved HF self-care adherence (Alvarez et al., 2016; Celano et al., 2018, 2020; Meraz et al., 2023c,d). Studies have shown that positive affect, especially gratitude, increases patients’ willingness to engage in HF self-care and supports their resilience when facing challenges (Celano et al., 2020; Meraz et al., 2023c,d). A PTG-based intervention that emphasizes appreciation of life and cultivates gratitude may help patients with positive reframing to reduce distress and improve motivation for self-care adherence.

### 3.5 New possibilities and adaptive coping

When patients struggle with HF self-care, it can feel like a cycle of stress and failure, making it difficult to imagine new ways of meeting their self-care goals (Meraz et al., 2023d). The coping styles of patients with HF when they experience self-care-related stress have a substantial impact on adherence (Riegel et al., 2022). Avoidance coping behaviors significantly undermine self-care adherence (Shimode et al., 2025), whereas emotion-focused coping that incorporates acceptance and problem-solving approaches enhances self-care adherence (Li and Shun, 2016). Our prior work shows that patients frequently perceived diuretic therapy as the most burdensome aspect of HF self-care; yet patients who employed adaptive coping strategies—planning ahead, maintaining flexibility, creating new routines—were more likely to adhere despite the challenges (Meraz et al., 2023d, 2025). Aligning with the new possibilities domain of PTG, an intervention that can teach acceptance, problem-solving, planning, insight, and flexibility can promote adaptive coping to HF self-care challenges, enabling an openness to new routines and self-care goals.

### 3.6 PTG framework for heart failure intervention design: the CardioWell model

**Figure 1:**
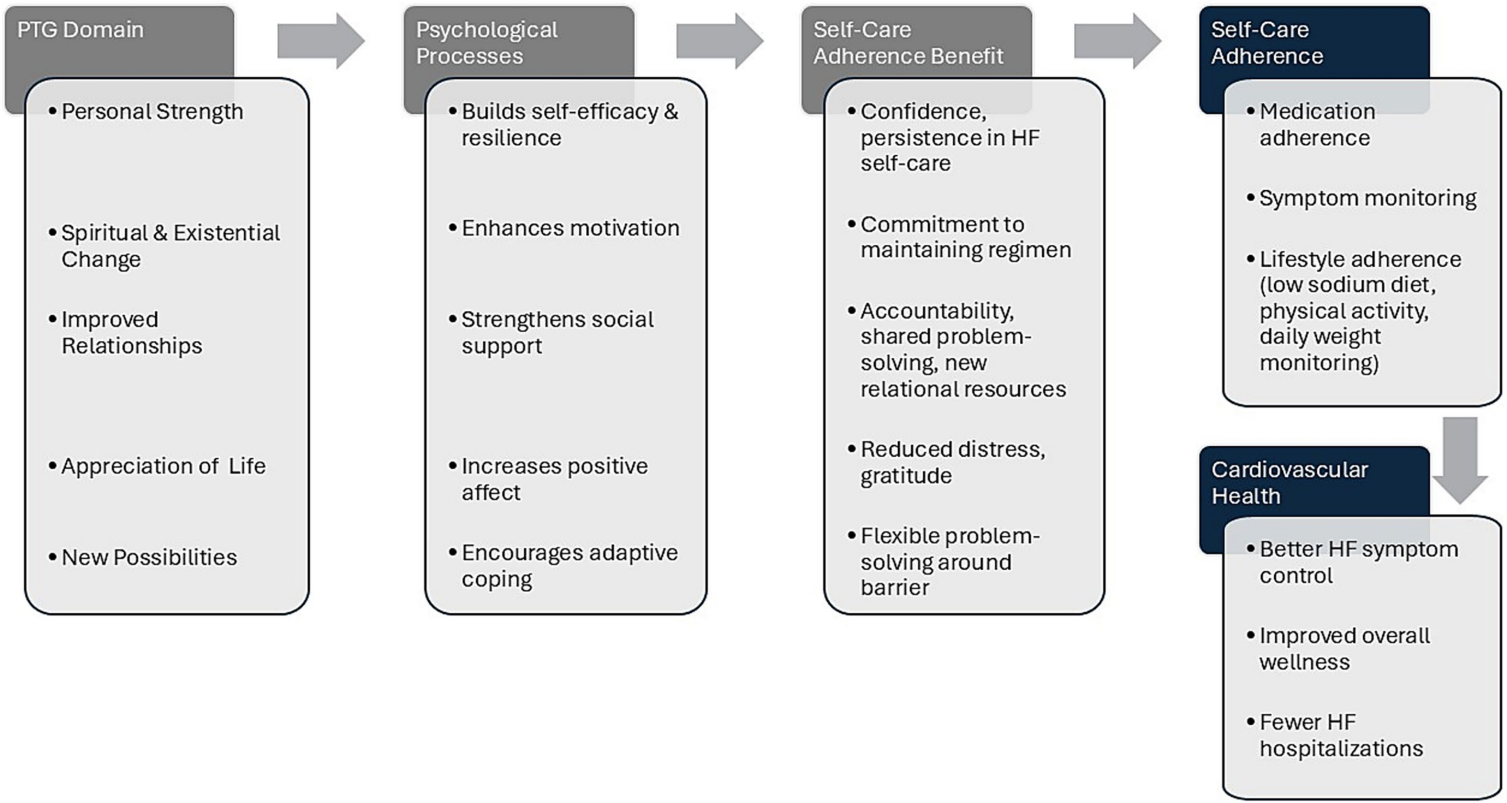
A conceptual model for PTG-Informed intervention design.

We propose that PTG theory offers a useful framework for growth-oriented interventions targeting HF self-care adherence. CardioWell (see Figure 1) is presented here as a theory-informed model that illustrates how PTG principles could be operationalized to support HF self-care adherence and to serve as a foundation for intervention design. The conceptualization of CardioWell emerged from our exploratory analysis of participant narratives, through which PTG-related processes became visible. Further theory-driven analysis helped identify psychologically meaningful targets for supporting HF self-care adherence. The CardioWell intervention is envisioned as a structured, growth-oriented program that incorporates PTG-informed psychoeducation, guided reflection, and supportive dialogue focused on psychological growth in the context of HF self-care. The model includes a series of proposed weekly telehealth sessions and self-guided activities intended to facilitate reflection on personal strengths, meaning-making, gratitude, optimism, deeper relational connections, and adaptive coping. Thus, the primary contribution of this work is the introduction of PTG as a novel theoretical lens to HF self-care adherence, with the CardioWell conceptual model serving as a concrete operationalization of that lens. These components are intentionally oriented toward the documented psychological challenges that undermine self-care adherence, positioning growth-oriented processes as potential mechanisms to support sustained adherence to HF self-care behaviors.

The PTG framework offers several operational and practical advantages for HF self-care intervention design. The PTG framework is a structured, theory-based approach that not only emphasizes building psychological resources but also targets psychological change with the potential to support lasting adherence. Across the five PTG domains, growth-oriented experiences may influence adherence through several psychological mechanisms, including strengthened identity and self-efficacy, renewed meaning and purpose in self-care adherence, enhanced relational connections, increased positive affect and gratefulness, and greater coping flexibility. There are key intervention elements to target within each domain, such as meaning-making exercises, gratitude practices, strength-based reflections, social connection activities, and coping flexibility training with problem-solving activities to overcome barriers to adherence. The two illustrative cases that follow demonstrate how PTG-like processes were recognized in HF self-care experiences and how these insights informed the pathways represented in the CardioWell model.

## 4 Illustrative cases

To illustrate how PTG theory could function as a novel theoretical lens for HF self-care adherence, we draw on two theoretically rich cases from an early-stage positive psychology program development study that was not based on PTG theory. This formative study aimed to design, refine, and produce program materials for a six-week telehealth positive-psychology program focused on HF self-care adherence and resilience. The development sample was intentionally small, with four adults with HF plus two stakeholder interviews. Participants engaged in a 6-week series of prototype intervention sessions, during which they provided weekly iterative feedback on content, delivery, and usability, and then completed a semi-structured exit interview. All sessions and interviews were audio-recorded, transcribed verbatim, and uploaded into NVivo (Lumivero, 2024). Informed consent was obtained before any study procedures. The narratives included in this paper originate from qualitative data collected throughout the study.

A qualitative content analysis approach was used, incorporating both inductive and deductive methods (Assarroudi et al., 2018). In the inductive phase, PTG-related insights first emerged organically from the narratives, promoting us to consider PTG as a relevant interpretive lens. These emergent insights informed a subsequent deductive phase in which we examined participants’ reflections through the lens of PTG theory. The five PTG domains served as a guiding framework for identifying illustrative instances of personal strength, spiritual and existential change, appreciation of life, improved relationships, and new possibilities as they relate to HF self-care. The analytic goal was conceptual illustration to support the extension of theory rather than thematic saturation or hypothesis testing.

For this present paper, two cases were selected because their narratives contained particularly clear examples of PTG-like growth processes in HF self-care. These cases serve as conceptual illustrations of how PTG theory may be meaningfully extended to daily HF self-care. These examples also help clarify the proposed pathways in the CardioWell model.

### 4.1 Case example: Ms. A

Ms. A, a 56-year-old Black woman living with HF, was renting a single room in a private home. She was unmarried, lived alone, and reported limited social support from friends and family. She reported some college and an annual income of less than $20,000. She was disabled due to multiple comorbidities, including diabetes, hypertension, depression, disrupted sleep, and morbid obesity. At the start of the intervention, Ms. A described feeling isolated in her illness experience, noting that the demands of daily HF self-care often felt overwhelming and discouraging. Adherence to self-care was difficult for her, especially maintaining exercise, a low-salt diet, and consistent diuretic use. She described HF self-care as “a real struggle,” which left her feeling burdened and under significant stress.

During the interview, Ms. A reflected on positive aspects of her life. Reflecting upon past successes helped her rediscover inner strengths and the possibility for future successes in performing self-care tasks, saying, “I have done hard things before. I know I can do this [self-care].” She identified small but meaningful sources of gratitude, such as having a safe space to live, medications that help her feel well, and people who help her in little ways. She described how noticing simple things improved her mood, saying, “Now [after completing the intervention], when I get down, I purposely look for the sun, hear the birds sing.”

Ms. A reported that meaning-making and gratitude exercises made her feel optimistic about feeling well and breathing better, which, in turn, enhanced her motivation to adhere to her medication. She reflected on her successes by saying, “Been feeling better these days because I am better at taking my medications.” Her reflections suggested an emerging sense of meaning in HF self-care adherence and a growing ability to reframe HF management as purposeful rather than burdensome. As her perspective shifted, she also reported an increased sense of confidence in performing self-care tasks. This led to improved problem-solving around barriers to adherence, better consistency with her diuretic regimen, and the establishment of new personal health goals. She stated,

I realized that I am strong and able to do the things I did not like doing, such as taking my fluid pill. I made my own little bathroom station. It motivates me where I know I have to get the fluid off my heart and now I feel like I can make it to the bathroom in time. I am more mindful of what I need to do to stay healthy. Be aware of my body. No one else can do that but me. I have been taking my fluid pill everyday now.

While her external support system remained limited, her reflections suggested that an enhanced awareness of helpful people was motivation for sustaining self-care adherence: “I was reminded to notice and be thankful for the great people around me who help me. It makes my mood better and keeps me going.”

### 4.2 Case example: Ms. B

Ms. B was a 68-year-old White woman with HF, who was widowed and lived alone. Her highest level of education was high school, and she reported an annual income of less than $20,000. She lived with multiple comorbidities, including reduced mobility, depression, and disrupted sleep. At the beginning of the intervention, Ms. B described feeling isolated and incapable of consistently performing self-care practices, noting that her physical limitations and low mood often left her without the energy or motivation to manage her condition. She described HF self-care as “hard” and reported poor adherence to self-care practices, particularly with exercise, keeping doctor appointments, and following a low-salt diet.

Participating in gratitude practices gradually shifted her outlook, helping her feel more optimistic and more capable of engaging in self-care practices such as daily exercise. She also said that she had become more aware of her thoughts and actions, noting, “I am not one to smile a lot. But I have been smiling more lately.” She expressed a deep appreciation for her doctors and the medical care she received, as well as for her faith in God, which she described as a source of strength and comfort. As her perspective shifted, Ms. B was able to set new, realistic exercise goals that she felt confident pursuing, and she began to see new possibilities for improving her health and quality of life. She described an increased sense of flexibility when faced with setbacks and a renewed purpose to keep going, noting that challenges felt less overwhelming and more manageable. She reflected on her new exercise goals by saying:

What if everything changes and what if nothing changes. Sometimes you just have to step out…Maybe I have made this goal in the past and not followed it. Every day is a new day. Just because I messed up in the past, there are things that I can do to overcome the challenges. Made me think about the things I can do to overcome my challenges. Sometimes hard to remember…take a deep breath and go on. Even if things aren’t going right, go on.

As her motivation increased, so did creative problem-solving for HF self-care adherence, such as exercising while seated on the days she felt unwell. By the end of the program, Ms. B described a new sense of accountability and determination: “It’s helped with accountability. Do not want to leave yourself hanging,” and she concluded with a forward-looking reflection, “I feel like I can keep going even after the study is over.”

### 4.3 Cross-case insights by PTG domain

The illustrative cases contained examples of positive psychological growth processes that aligned with the five PTG domains. The following discussion highlights the ways in which these examples map onto PTG domains and thereby inform the theoretical pathways proposed in the CardioWell model.

In the *Personal Strength* domain, the two illustrative examples had examples of renewed confidence and resilience in managing HF self-care challenges. Consistent with PTG in the *Spiritual and Existential* domain, both example cases exhibited an expanded sense of meaning and purpose in adhering to self-care. The cases contained examples of leaning on spiritual faith and personal desire to feel well, transforming the adversity of adhering to stressful self-care tasks, such as exercising and taking diuretics, into a deepened sense of meaning and purpose. The subsequent gains in meaning and confidence led to viewing each act of adherence as an attainable and purposeful step toward better health and well-being.

Across cases, gratitude played a key role in fostering positive psychological change, consistent with the *Appreciation of Life* domain. In each case, gaining a deeper sense of gratitude for simple pleasures such as walking to the mailbox, sunshine, and even for HF medications once viewed as burdensome, improved mood and motivated better self-care adherence. In the *Improved Relationships* domain, gratitude also permeated relational growth, fostering a greater awareness of and appreciation for doctors and people who were resources for navigating self-care struggles. Taken together, gratitude not only supported participants in reframing the burden of HF self-care into a source of motivation but also served as a bridge across PTG domains, linking appreciation of life with enhanced purpose and relationships in ways that reinforced positive psychological growth, coping, and adherence.

The two illustrative cases contained examples that aligned with the *New Possibilities* domain. Together, an enhanced sense of confidence, purpose, and gratitude helped them recognize what was possible and to develop new health goals and strategies for adherence. Enhanced adaptive coping, such as flexibility with changing circumstances, creative problem-solving, and acceptance of the reality of self-care challenges, helped participants manage self-care-related stress and find new avenues for adherence.

## 5 Discussion

In this paper, we propose that PTG theory can be meaningfully extended to HF self-care and present a PTG-informed conceptual model for designing interventions to support self-care adherence. Drawing on insights from two illustrative cases, the following discussion considers how growth-oriented psychological processes may be engaged in response to the ongoing demands of HF self-care and how these processes informed the conceptualization of the CardioWell intervention model (Figure 1). We further offer a conceptual explanation for the pathways represented in the CardioWell model by outlining how each PTG domain may activate distinct psychological processes that could support HF self-care adherence.

The illustrative cases provided theoretically rich examples of how patients who were struggling with HF self-care adherence engaged in PTG-related cognitive and emotional processes, including meaning-making, strengthened personal agency, gratitude building, relational awareness, and flexible coping, which appeared to support their ability to adapt to self-care demands in ways that supported improved adherence. The illustrative cases, when viewed from an intervention design perspective, clarify how PTG processes may emerge in response to adherence-related challenges and inform the pathways depicted in the CardioWell model. Specifically, the cases highlighted the potential for self-care-related adversity and distress to serve as catalysts for constructive reflection, positive reframing of self-care tasks, and an enhanced sense of purpose, appreciation, and adaptive coping. PTG theory is particularly well-suited in this context because it accounts for how disruption, struggle, and adaptation can foster growth-oriented processes that influence motivation, persistence, and engagement over time. By integrating PTG theory with illustrative case insights, this work extends PTG into the area of HF self-care and provides a theoretically grounded rationale for the design of CardioWell as a growth-oriented intervention model to support HF self-care adherence.

Although PTG shares conceptual space with other positive psychological approaches, it brings a distinctly different perspective to HF self-care. Most resilience-focused interventions aim to help people “bounce back” or hold steady in the face of hardship (Liu et al., 2020). PTG, by contrast, centers on the potential for genuine transformation that grows out of the struggle itself, which is particularly relevant to the identity upheaval and existential questions so many people with HF experience (Tedeschi and Calhoun, 2004). Meaning-centered approaches highlight purpose and values in illness (Park et al., 2019), but PTG goes further by integrating meaning-making with other powerful domains, such as personal strengths, deepening relationships, finding appreciation, and openness to new possibilities. Similarly, many general positive psychology-based programs target discrete strengths, such as gratitude or optimism (Chakhssi et al., 2018), whereas PTG offers a more comprehensive and theoretically coherent framework that explains how adaptation and growth can emerge across multiple domains as individuals work to cope with the ongoing challenges of chronic illness. Importantly, PTG focuses on deeper, more enduring psychological shifts that may help support long-term adherence. Most positive psychology interventions primarily aim to improve mood and well-being rather than directly targeting health behaviors (Feig et al., 2022). The few studies have shown mixed effects on health behaviors, with only a few fully powered trials reaching statistical significance (Feig et al., 2022). Together, these distinctions highlight the added value of PTG as a guiding framework for intervention design, offering a growth-oriented approach that is conceptually aligned with the ongoing struggle inherent in adhering to HF self-care tasks.

### 5.1 Proposed pathways linking PTG to self-care adherence and implications for intervention design

The CardioWell model offers a practical conceptual foundation for future intervention design by outlining potential PTG-related pathways that may activate specific psychological processes that, in turn, benefit known determinants of HF self-care adherence. Organized by the five PTG domains, these pathways articulate how growth-oriented processes may benefit self-care adherence (i.e., consistent medication adherence, routine symptom monitoring, dietary regulation, regular physical activity, and timely symptom reporting). Ultimately, sustained self-care adherence could lead to greater clinical stability and better HF outcomes.

We propose that growth in personal strength may enhance self-efficacy, resilience, and perceived capability, which are central drivers of self-care adherence (Fivecoat et al., 2018; Huang et al., 2022; Meraz et al., 2023b). As patients gain confidence and begin to view themselves as capable and resilient, they may be more persistent in tough medication-taking routines and self-care tasks. Spiritual and existential changes may help patients rediscover meaning and commitment to self-care regimens, transforming self-care from a burdensome obligation into a purposeful act. This shift may increase intrinsic motivation and support sustained engagement in demanding tasks such as exercise and diuretic adherence (Sacco et al., 2019; Meraz et al., 2023c).

Relational growth may increase perceived social support, open new avenues for relational resources, or reduce isolation, all of which are known predictors of adherence (Fivecoat et al., 2018; Irani et al., 2019; Meraz et al., 2023b). Patients who feel supported are often more willing to seek help with self-care tasks, communicate symptoms early, and maintain self-care routines. Greater appreciation of life may elevate positive affect and foster gratitude, both of which have been linked to better adherence (Ferguson et al., 2017; Meraz et al., 2023d; Rashid et al., 2023). Positive emotional states can increase motivation and have been shown to predict better self-care adherence. Recognizing new possibilities may strengthen coping flexibility and problem-solving, helping patients develop new ways to meet their goals when they face setbacks (Riegel et al., 2022). This can lead to using practical adherence strategies, such as planning ahead, creating new habits, or adjusting self-care routines on difficult days. Together, these proposed mechanisms show how PTG-related processes could be intentionally targeted in an intervention to shape daily decisions that determine long-term adherence to HF self-care.

To illustrate how the PTG domains could be operationalized, the CardioWell intervention could be a brief, structured telehealth program. This program would include six weekly sessions lasting 45 to 60 min each, supplemented by simple at-home reflection activities. Each session would introduce a PTG domain with brief psychoeducation, followed by guided reflection exercises and application to a real self-care challenge. For example:

- In the personal strength session, patients might recall times they have overcome difficulty in the past and connect that inner strength to adhering to challenging self-care tasks.
- The meaning and existential session could invite patients to reflect on what matters most to them and why continuing self-care is personally meaningful.
- The relational session might incorporate mapping supportive relationships and practicing communication strategies for seeking help.
- The appreciation of life session could include simple gratitude or savoring exercises to ease the distress around burdensome tasks.
- And the new possibilities session could walk patients through flexible problem-solving and setting small, realistic new goals when self-care routines do not go as planned.

These examples show how PTG principles can be translated into realistic PTG-based activities and demonstrate how the CardioWell model could be developed and tested in future intervention work.

### 5.2 Future research directions

This work represents an early-stage, theory-generating effort intended to inform intervention conceptualization rather than address feasibility or acceptance, test intervention effects, or establish generalizations. The illustrative cases exemplified how PTG-related processes may emerge in the context of HF self-care and informed the development of the CardioWell conceptual model. As such, the proposed pathways should be empirically evaluated in future research. Although the homogeneous sample was sufficient for conceptual work, future research should formally evaluate PTG-informed interventions in larger, more diverse samples, assess mechanistic pathways, and evaluate their effects on sustained self-care adherence and clinical outcomes. Because PTG theory has not been applied in HF contexts, additional work is also needed to refine and validate measures of PTG-related processes in chronic illness and assess the feasibility, acceptability, and cultural responsiveness of PTG-based intervention components. These steps are essential for advancing PTG from a promising conceptual framework to an empirically supported approach for improving HF self-care.

## 6 Conclusion

This paper advances PTG theory as a novel conceptual framework for understanding positive psychological change in the context of HF self-care and for informing the development of growth-oriented self-care interventions. Drawing from two illustrative cases from early-stage intervention work, we showed how participants’ experiences reflected processes aligned with PTG domains, including personal strength, spiritual and existential change, improved relationships, appreciation of life, and new possibilities. These growth-oriented processes appeared to support coping with self-care adversity in ways that benefited adherence. By identifying psychologically meaningful targets and proposing conceptual pathways linking PTG-related processes to HF self-care, this work provides a strong theoretical foundation for the PTG-informed CardioWell model. However, these pathways remain exploratory. Future research is needed to systematically develop and test PTG-informed interventions in larger and more diverse HF populations to evaluate their potential impact on long-term self-care adherence and cardiovascular outcomes. Overall, this paper supports extending PTG theory to HF self-care research and highlights its promise as a framework for understanding and addressing the psychological processes that shape adherence in chronic illness management.

## Data Availability

The raw data supporting the conclusions of this article will be made available by the authors, without undue reservation.
